# CYP51 is an essential drug target for the treatment of primary amoebic meningoencephalitis (PAM)

**DOI:** 10.1371/journal.pntd.0006104

**Published:** 2017-12-28

**Authors:** Anjan Debnath, Claudia M. Calvet, Gareth Jennings, Wenxu Zhou, Alexander Aksenov, Madeline R. Luth, Ruben Abagyan, W. David Nes, James H. McKerrow, Larissa M. Podust

**Affiliations:** 1 Center for Discovery and Innovation in Parasitic Diseases, Skaggs School of Pharmacy and Pharmaceutical Sciences, University of California San Diego, La Jolla, California, United States of America; 2 Cellular Ultrastructure Laboratory, Oswaldo Cruz Institute, FIOCRUZ, Rio de Janeiro, RJ, Brazil; 3 Department of Chemistry & Biochemistry, Texas Tech University, Lubbock, Texas, United States of America; University of Tennessee, UNITED STATES

## Abstract

Primary Amoebic Meningoencephalitis (PAM) is caused by *Naegleria fowleri*, a free-living amoeba that occasionally infects humans. While considered “rare” (but likely underreported) the high mortality rate and lack of established success in treatment makes PAM a particularly devastating infection. In the absence of economic inducements to invest in development of anti-PAM drugs by the pharmaceutical industry, anti-PAM drug discovery largely relies on drug ‘repurposing’—a cost effective strategy to apply known drugs for treatment of rare or neglected diseases. Similar to fungi, *N*. *fowleri* has an essential requirement for ergosterol, a building block of plasma and cell membranes. Disruption of sterol biosynthesis by small-molecule inhibitors is a validated interventional strategy against fungal pathogens of medical and agricultural importance. The *N*. *fowleri* genome encodes the sterol 14-demethylase (CYP51) target sharing ~35% sequence identity to fungal orthologues. The similarity of targets raises the possibility of repurposing anti-mycotic drugs and optimization of their usage for the treatment of PAM. In this work, we (**i**) systematically assessed the impact of anti-fungal azole drugs, known as conazoles, on sterol biosynthesis and viability of cultured *N*. *fowleri* trophozotes, (**ii**) identified the endogenous CYP51 substrate by mass spectrometry analysis of *N*. *fowleri* lipids, and (**iii**) analyzed the interactions between the recombinant CYP51 target and conazoles by UV-vis spectroscopy and x-ray crystallography. Collectively, the target-based and parasite-based data obtained in these studies validated CYP51 as a potentially ‘druggable’ target in *N*. *fowleri*, and conazole drugs as the candidates for assessment in the animal model of PAM.

## Author summary

The free-living amoeba, *Naegleria fowleri*, is commonly found in water sources including swimming pools having inadequate levels of chlorine, lakes, and rivers. *N*. *fowleri* can act as an opportunistic pathogen causing severe brain injury called Primary Amebic Meningoencephalitis (PAM), often in healthy children and young adults. With a fatality rate over 97%, *N*. *fowleri* is considered one of the most deadly human pathogens. Despite the fact that precious lives are lost annually, *N*. *fowleri* infection is rare and economic incentives to invest in development of anti-PAM drugs by the pharmaceutical industry are lacking. In the absence of economic inducements, drug ‘repurposing’ is a cost effective strategy for re-profiling known drugs for the treatment of neglected diseases. The similarity between pathogenic fungi and free-living amoebae in the sterol biosynthesis pathway encouraged us to assess anti-mycotic drugs, clinically-approved for treatment of a variety of fungal diseases, for anti-*Naegleria* activity. Carrying out biochemical studies and x-ray-crystallography of the target enzyme, we demonstrated that azole anti-fungal drugs, known as conazoles, disrupt sterol biosynthesis in amoebae by competing with the natural substrate for binding in the active site of the sterol 14-demethylase (CYP51). Disruption of sterol biosynthesis leads to a rapid death of pathogenic amoebae.

## Introduction

The amphizoic amoeba (existing both in free-living and parasitic forms), *Naegleria fowleri* is commonly found in water resources such as swimming pools having inadequate levels of chlorine, lakes and rivers. It feeds mostly on bacteria, but can also act as an opportunistic pathogen causing infection of the central nervous system (CNS) of humans and animals.[1] *N*. *fowleri* usually infects people when contaminated water enters the body through the nose. Following infection, *N*. *fowleri* infiltrates the nasal mucosa and passes along the olfactory neuroepithelial route to invade the brain. *N*. *fowleri* causes severe primary amebic meningoencephalitis (PAM) resulting in cerebral edema and destruction of brain tissue, mostly in healthy children and young adults.[2] PAM due to *N*. *fowleri* has a worldwide distribution although it occurs most frequently in tropical areas and during hot summer months.[3] Infection is considered rare in the United States (0–8 infections per year)[4] but PAM cases may go unnoticed among other infections, particularly in the developing countries.[1] Noteworthy, PAM is not on the National Notifiable Diseases Surveillance list; thus, reporting of the national incidence of PAM by the US Centers for Disease Control and Prevention (CDC) depends on individual state health departments to report diseases voluntarily. Despite modern improvements in antimicrobial therapy and supportive medical care, the fatality rate associated with *N*. *fowleri* PAM is >97%.[4] The disease is particularly problematic due to both its rapid onset and the lack of effective treatments.[5] Currently, there is no single, proven, evidence-based treatment with a high probability of cure. The full recovery of a patient in the summer of 2013, after 35 years without a *Naegleria* survivor in the US, was attributed to early diagnosis and treatment, and the use of combination therapy including the investigational drug miltefosine and induced hypothermia.[6] In the absence of data to estimate the true risk of PAM or to set up and reinforce the measurable standards to protect the human population, early diagnosis and aggressive antimicrobial treatment remain the only option to treat the disease.

The CDC-recommended treatment for patients suspected of PAM currently includes combination therapy consisting of anti-mycotic drugs amphotericin B (AmpB) and fluconazole, antibiotics azithromycin and rifampin, the investigational anti-cancer agent miltefosine and, finally, an anti-inflammatory drug, dexamethasone, to reduce the cerebral edema.[1] AmpB, a cornerstone of PAM therapy and a standard of care for CNS infections caused by molds, acts via binding ergosterol in cell membranes causing rapid leakage of monovalent ions leading to cell death.[7] Clinical use of AmpB is limited due to its toxicity, including acute infusion-related reactions and dose-related nephrotoxicity.[1] Fluconazole is another anti-mycotic drug that acts via a different mechanism. Fluconazole depletes the ergosterol pool by blocking removal of the methyl group at C-14 position of a biosynthetic precursor catalyzed by sterol 14-demethylase (CYP51).[8–11] Fluconazole belongs to the ‘conazole’ pedigree of antifungal agents targeting CYP51.[12] This drug class also includes miconazole, ketoconazole, voriconazole and itraconazole that were previously reported to exhibit amoebicidal effect against *N*. *fowleri in vitro*.[13–17] Fluconazole and miconazole have also been used in combination with other drugs as part of treatment regimens in human PAM patients.[6, 18, 19]

D*e novo* sterol biosynthesis from squalene takes place in most high eukaryotes (although it is lost in certain lineages, *e*.*g*. insects and worms) and also in lower eukaryotes with an aerobic life style.[20] The latter include human pathogens such as fungi, kinetoplastids and free-living amoebae. Disruption of sterol biosynthesis by small-molecule inhibitors is a validated interventional strategy against fungal pathogens of medical and agricultural importance. CYP51 is one of the most extensively exploited drug targets for the development of anti-fungal agents. Kinetoplastids are major parasite targets for the development of CYP51 inhibitors outside traditional antifungal drug discovery programs.[21]

Similar to fungi and kinetoplastids, amoebae from the genera *Naegleria* and *Acanthamoeba* have an essential requirement for ergosterol.[22–24] In contrast to the lanosterol route in fungi and kinetoplastids,[25] biosynthesis of ergosterol in amoebae occurs via cycloartenol, a sterol biosynthetic precursor typical of photosynthetic organisms, ie., algae and plants.[22–24] *N*. *fowleri* genome encodes CYP51 (NfCYP51; AmoebaDB accession number NF0102700) sharing ~35% sequence identity to human, fungal and kinetoplastid orthologues. Higher sequence identity to plant (<40%), *Acanthamoeba* (42%), and a non-pathogenic *Naegleria gruberi* (86%) is consistent with the ‘plant-like’ substrate specificity of NfCYP51.[23] The similarity of targets raises the possibility of ‘repurposing’ anti-mycotic drugs, clinically-approved against a variety of fungal diseases, and optimization of azole-based PAM therapy for treatment of PAM.

In this work, we (**1**) chemically validate sterol biosynthesis pathway as a “druggable” target in *N*. *fowleri* and (**2**) systematically assess the efficacy of the anti-fungal azole drugs, known as conazoles (including the latest additions to the armamentarium of the anti-fungal drugs, posaconazole and isavuconazole) versus both the whole organism and the recombinant molecular target. In the course of the studies, we (**i**) validated NfCYP51 as an essential biosynthetic enzyme in *N*. *fowleri*, (**ii**) determined the endogenous NfCYP51 substrate, and (**iii**) characterized drug-target interactions by UV-vis spectroscopy and protein x-ray crystallography.

## Results and discussion

### Anti-proliferative effect of conazoles *in vitro*

**Fig 1:**
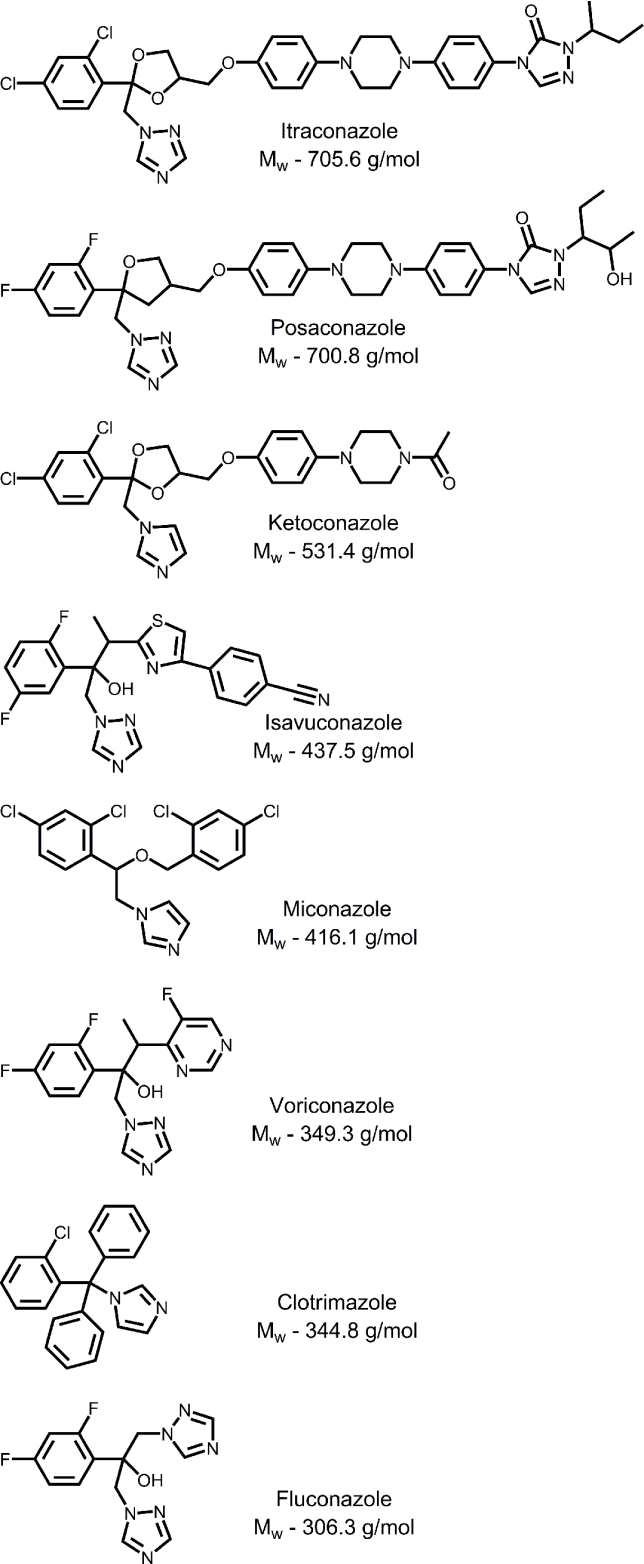
Azole antifungal drugs also known as conazoles.

An assay previously developed and validated [26] was used to assess anti-proliferative activity of the conazoles presented in **Fig 1**. In this assay, conazoles demonstrated anti-*N*. *fowleri* activity in a broad range of concentrations—from an EC_50_ of 13.9 μM (fluconazole) to ≤0.01 μM (for itraconazole and posaconazole) (**Table 1**). Notably, *in vitro* potency of all conazole drugs exceeded that of miltefosine; itraconazole and posaconazole were an order of magnitude more potent than AmpB, while ketoconazole and isavuconazole were equipotent to AmpB. Across the board, anti-*Naegleria* potency of conazoles increased with the increase of the molecular weight (MW), from fluconazole (306.3 g/mol) to itraconazole (705.6 g/mol). Between 340 g/mol and 450 g/mol, anti-proliferative activity of conazoles correlated with lipophilicity per non-hydrogen atom expressed as logP/MW values—more lipophilic drugs demonstrated higher potency (**Table 1)**. This trend is explained by the hydrophobicity of the NfCYP51 binding site favoring binding of lipophilic molecules, as demonstrated by the co-crystal structures determined in this work. The potency observed for fluconazole (EC_50_ of 13.9 μM, equivalent of 4.3 mg/ml) was lower than the MIC_50_ values of 0.5–2.0 mg/ml reported elsewhere for *N*. *fowleri* isolates, either from natural water sources[16] or from patients who had died of PAM.[15]

**Table 1:** Inhibition of *N*. *fowleri* with conazoles.

Drugs	MW, g/mol	logP	logP/MW, ×10^3^	logD at pH 7.4	EC_50_, μM
Itraconazole	705.6	5.7^a^	8.1	>5^b^	≤0.01
Posaconazole	700.8	5.5^a^	7.8	2.15^b^	≤0.01
Ketoconazole	531.4	4.4^a^	8.3	3.7[27]	0.1±0.04
Isavuconazole	437.5	3.1^c^	7.1	3.1^3^	0.1±0.04
Miconazole	416.1	6.1^a^	14.6	6.3[28]	2.0±0.04
Voriconazole	349.3	1.0^a^	2.9	1.8^b^	76% at 25 μM
Clotrimazole	344.8	6.1^a^	17.7	5.2[29]	0.6±0.03
Fluconazole	306.3	0.4^a^	1.3	0.5^b^	13.9±0.01
**Standards of care**					
Amphotericin B	924.1	0.8^a^		-2.8^b^	0.1±0.01
Miltefosine	407.6	3.4^c^		4.0^c^	54.5±0.01

^a^experimental logP values, as reported by the Drug Bank (www.drugbank.ca)

^b^Reviewed elsewhere[30]

^c^calculated logP and logD values are from EMBL-EBI (www.ebi.ac.uk)

### Growth inhibition as a function of time

**Fig 2:**
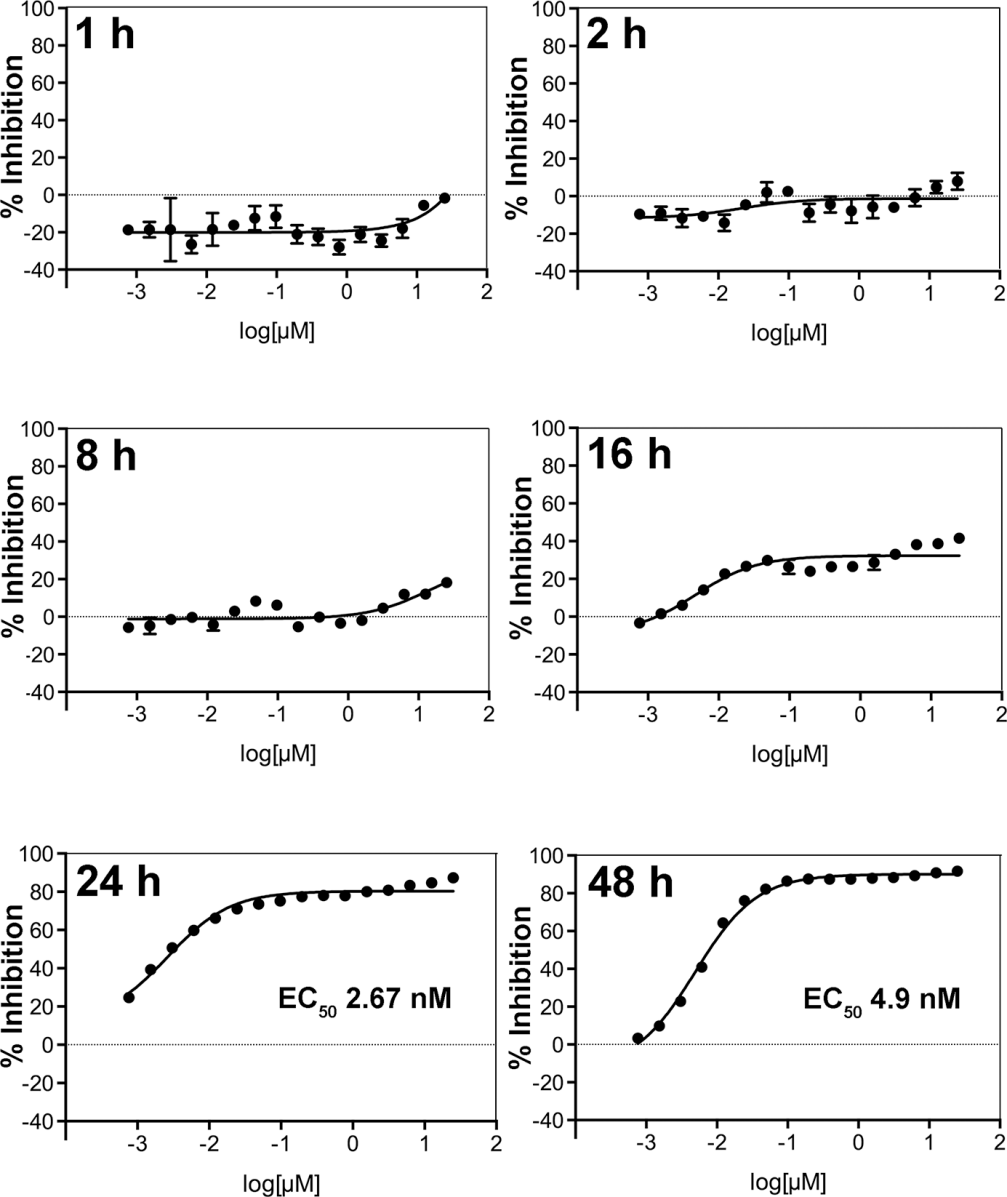
Posaconazole growth inhibition curves at different time points.

To answer the question of how fast CYP51 inhibitors kill *N*. *fowleri*, we measured growth inhibition dose-response to posaconazole at different time points. *N*. *fowleri* trophozoites were exposed to a single dose of posaconazole serially diluted in 96-well format from 25 μM to 0.008 μM in 0.2% sulfobutylether-β-cyclodextrin (SBE-β-CD), also known as Captisol. SBE-β-CD is used as an excipient (a formulating agent) to increase the solubility of poorly soluble drugs, including posaconazole in the Noxafil intravenous formulation (Merck).[31] Growth inhibition curves constructed for different time points (**Fig 2**) demonstrate an inhibitory effect of posaconazole at the highest concentrations as early as 8 h post-exposure. Inhibition reaches 40% at 16 h post-exposure, ~90% at 24 h (EC_50_ of 2.7 nM) and maximizes at 48 h (EC_50_ of 4.9 nM).

### Target engagement in *N*. *fowleri* trophozoites

#### Impact of posaconazole on sterol biosynthesis

**Fig 3:**
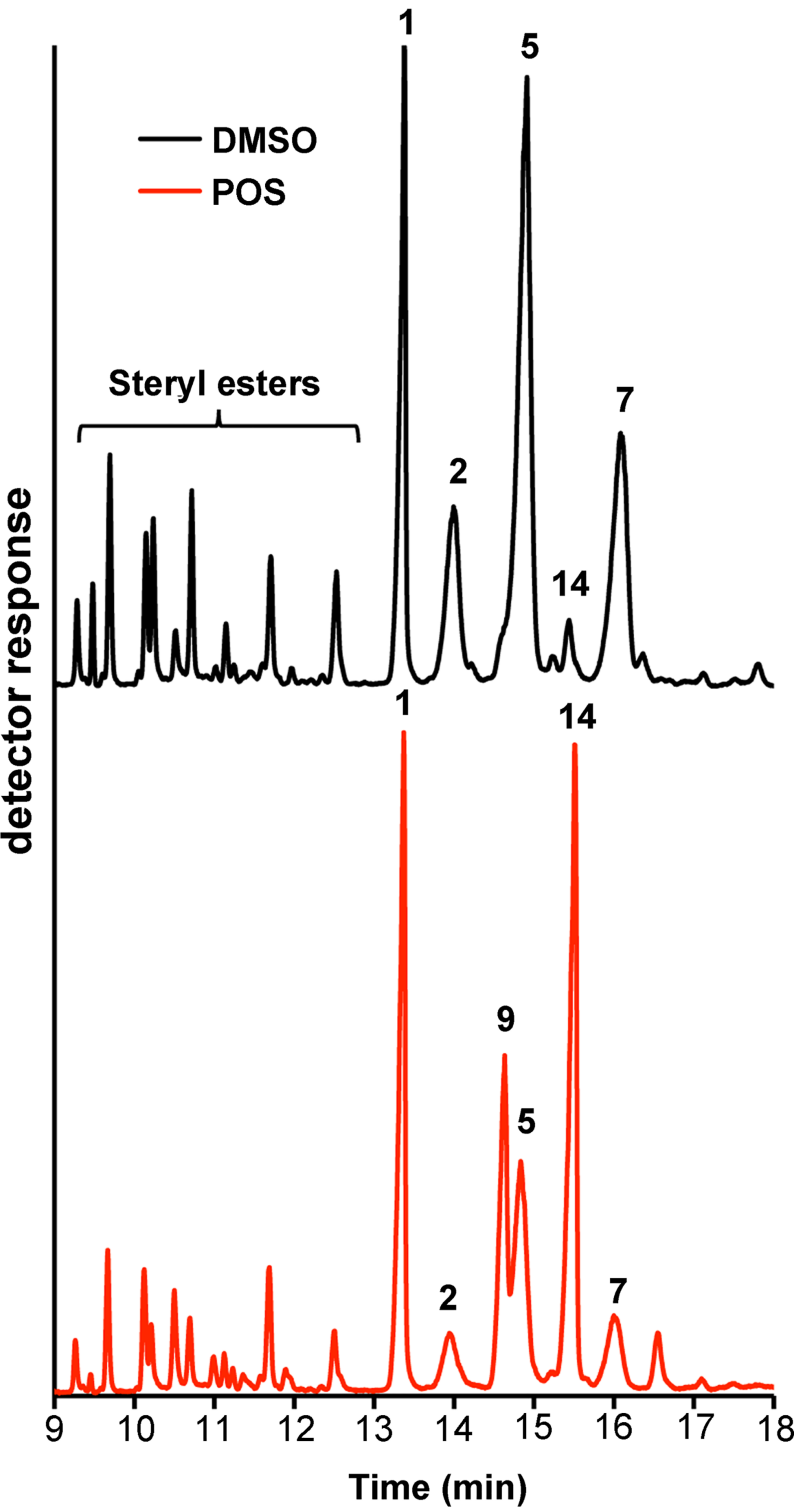
Gas chromatography separation of the total sterol fractions extracted from *N*. *fowleri* trophozoites. Chromatogram fragments from the non-derivatized DMSO- (*black trace*) and posaconazole (POS)-treated (*red trace*) sterol extracts are shown. Peaks are labeled according to **Table 2**. The content of 31-norlanosterol (**14**) remarkably increased in posaconazole-treated samples.

To chemically validate NfCYP51 as a therapeutic drug target, we analyzed metabolites accumulated in *N*. *fowleri* in response to posaconazole. Lipids extracted from 20 or 50 million trophozoites treated with 0.1% DMSO, 0.2 μM AmpB or 0.2 μM posaconazole for 24 hours were subjected to gas chromatography-mass spectrometry (GC-MS) analysis. The sterol identities were assigned based on relative chromatographic behavior, the characteristic molecular masses and electron ionization (EI) fragmentation patterns of free sterols (**Fig 3**) or of the trimethylsilyl (TMS)-derivatized sterols (**S1 Fig**).

**Fig 4:**
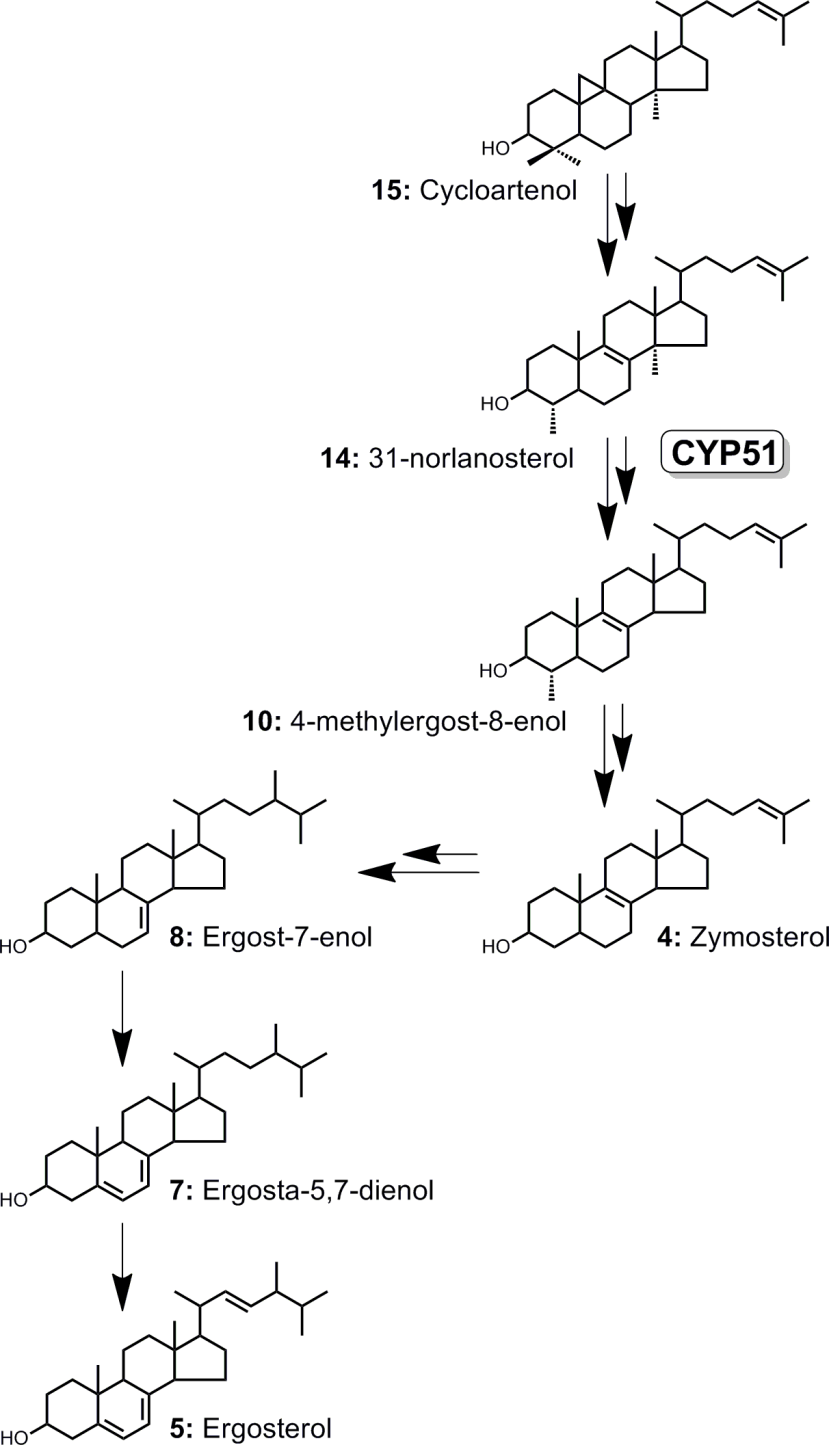
The mainstream of the ergosterol biosynthesis cascade in *N*. *fowleri*. Sterols are numbered according to the **Table 2**.

Similar to other previously characterized free-living amoebae of *Naegleria* and *Acanthamoeba* genera,[22–24] sterol biosynthesis in *N*. *fowleri* proceeds from cycloartenol (**15**) via 31-norlanosterol (**14**) to ergosterol (**5**) (**Fig 4**). Ergosterol and its biosynthetic precursor ergosta-5,7-dienol (**7**) are the two most abundant endogenous sterols dominating lipid extracts of DMSO-treated *N*. *fowleri* (48% of total sterol mass), whereas 4-monomethyl-, 4,4-dimethyl- and 14α-methylsterols are represented sparsely (**Table 2**). Cholesterol (**1**) constituting about 25% of sterol mass in both DMSO- and posaconazole-treated samples (**Table 2**), is likely ingested from the growth media containing fetal bovine serum (FBS) and liver extract. AmpB, a drug with a different mechanism of action used as a negative control, did not perturb the native sterol pattern (**S1 Fig**).

**Table 2:** Sterol composition in *N*. *fowleri* treated with 0.1% DMSO or 0.2 μM posaconazole.

Sterols		RTTc^a^	% Composition	
			DMSO	Posaconazole
	*4-Desmethylsterols (% of total)*		*90*.*0*	*50*.*5*
**1**	Cholesterol	1	25.6	26.1
**2**	7-Dehydrocholesterol	1.044	12.5	3.9
**3**	Lathosterol	1.061	0.8	
**4**	**Zymosterol**^**b**^	1.068	**0.4**	
**5**	**Ergosterol**	1.114	**26.7**	**14.2**
**6**	Ergost-5-enol	1.136	1.4	0.7
**7**	**Ergosta-5,7-dienol**	1.202	**21.3**	**5.7**
**8**	**Ergost-7-enol**	1.22	**1.2**	
	*4-Monomethylsterols*		*6*.*8*	*48*.*5*
**9**	4α,14α-Dimethylcholest-8-enol	1.082	1.1	15.5
**10**	**4α-Methylergost-8-enol**	1.095	**3.5**	**0.4**
**14**	**31-Norlanosterol**	1.143	**2.2**	**29.7**
**11**	Δ^7^-31-norlanosterol	1.238		2.9
	*4*,*4-Dimethylsterols*		*3*.*2*	*1*.*0*
**12**	24-Dihydrolanosterol	1.304	0.2	0.2
**13**	24-Dihydrocycloartenol	1.324	1.1	
**14**	Parkeol	1.384		0.1
**15**	**Cycloartenol**	1.406	2.0	0.7
	*14*α*-Methylsterols*		*6*.*5*	*49*.*1*
	*Δ*^*24(25)*^*-sterols*		*8*.*1*	

^a^relative retention time compared to cholesterol

^b^in bold are highlighted sterols constituting the mainstream of biosynthesis cascade in **Fig 4**

Upon treatment with posaconazole, the ratio of metabolites changed dramatically. The total content of the 14α-methylsterols increased from 6.5% to 49.1%, with the major accumulated intermediate being 31-norlanosterol, followed by its C24-C25 hydrogenated product, 4,14-dimethylcholest-8-enol (**9**) (**Table 2**). Removal of the 4β-methyl group was unaffected by posaconazole, meaning that it likely occurs prior to 14-demethylation. Based on these data, 31-norlanosterol serves an endogenous substrate of NfCYP51. Accumulation of the 4α-methylsterols in posaconazole-treated *N*. *fowleri*—increase from 6.8% to 48.5%—indicates that removal of the 4α-methyl group occurs downstream of CYP51. Finally, both C24 methylation catalyzed by sterol C24-methyltransferase, double bond isomerization catalyzed by sterol Δ^8^-Δ^7^-isomerase (reaction **4→8**), and C22-C23 bond desaturation catalyzed by Δ^22^-desaturase, also occur downstream of CYP51 (reaction **7→5**) (**Fig 4**).

#### Steryl esters

Along with the sterol intermediates, multiple steryl esters were detected in the lipid extracts (**Fig 3**). Steryl moieties were identified by the molecular masses calculated from the m/z values of the [M-ROH]^+^ fragments compared with the fragmentation patterns to the sterol acetate standards in the NIST database (**Table 3**). The fatty acid identities of steryl esters have not been analyzed due to limited sample size. Upon exposure to posaconazole, the steryl ester pool declined compared to the free sterols, as judged by the steryl ester/free sterol ratio (**Table 3**). The composition of the steryl ester pool also changed. The notable increase in cholesteryl ester from 4.3 to 13.6% may be an attempt to compensate for the deficit of endogenous sterols. On the contrary, the content of squalene dropped in the posaconazole-treated sample from 5.5 to 1.5% suggesting a regulatory loop signaling excess of the downstream intermediates.

**Table 3:** Composition of the steryl ester pool in *N*. *fowleri*.

Steryl ester^a^	[M-ROH]^+^	RT (min)	% of Total	
			DMSO	Posaconazole
7-Dehydrocholesteryl ester-1	366	9.3	6.1	5.7
Squalene^b^	410	9.5	5.5	1.5
Ergosteryl ester	378	9.7	15.9	16.1
5,7,24-Triene-ergosteryl ester-1	378	10.1	11.6	15.6
5,7-Diene-ergosteryl ester-1	380	10.2	13.0	8.2
Cholesteryl ester	368	10.5	4.3	13.6
5,7-Diene-ergosteryl ester-2	380	10.7	12.8	8.4
7-Dehydrocholesteryl ester-2	366	11.1	4.6	3.6
5,7,24-Triene-ergosteryl ester-2	378	11.7	12.6	18.2
5,7-Diene-ergosteryl ester-3	380	12.5	13.5	9.1
Ratio (steryl ester/free sterol)^c^			0.24	0.15

^a^The fatty acid moieties were not identified (the MS scan range was from 50 to 450 amu)

^b^Not a steryl ester.

^c^The ratio was calculated by dividing a sum of total ion count peak areas of the steryl esters by that of free sterols.

### Impact of posaconazole on the *N*. *fowleri* cell ultrastructure

**Fig 5:**
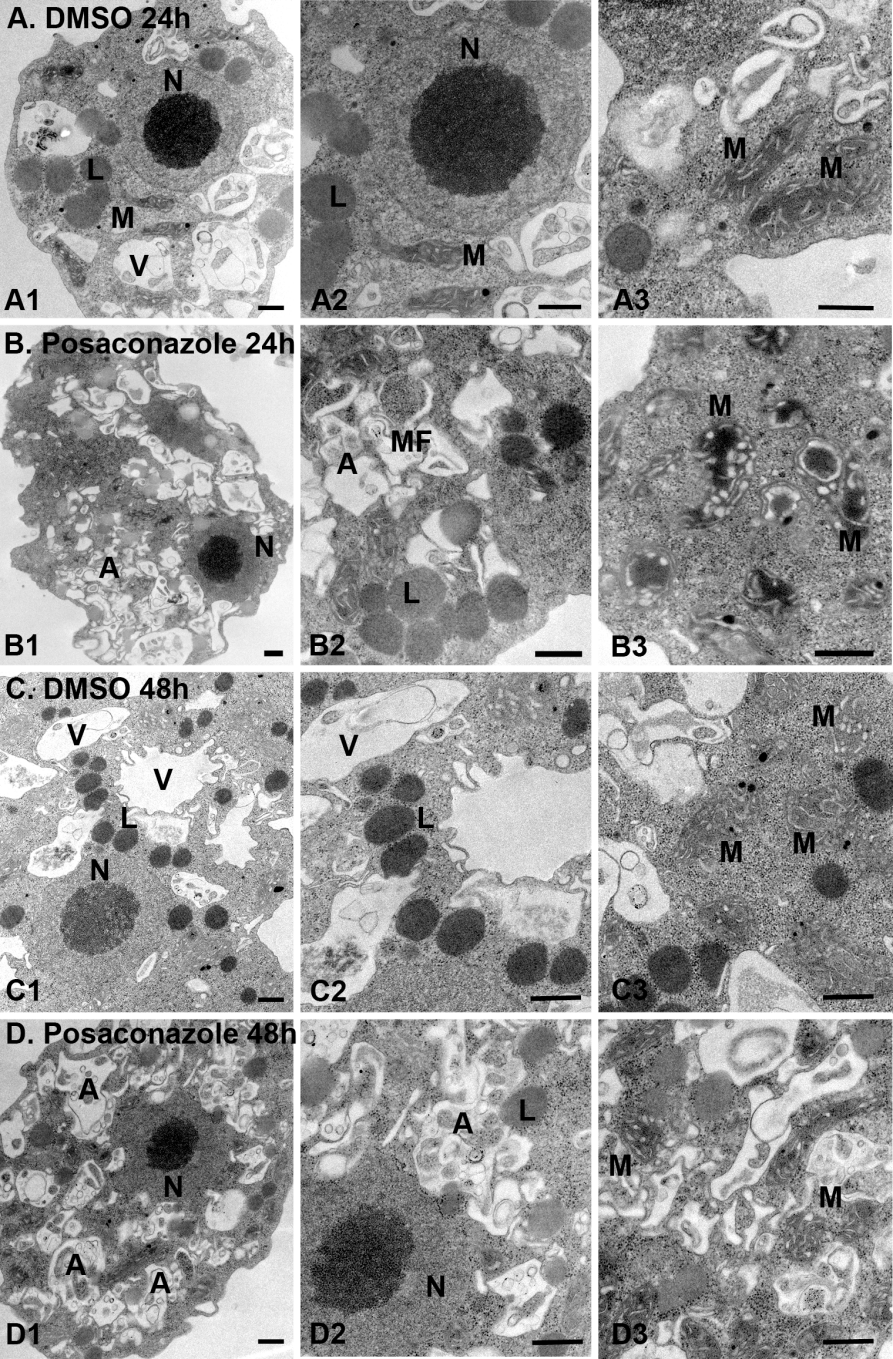
Ultrastructural analysis of posaconazole-treated *N*. *fowleri* by transmission electron microscopy (TEM). 0.1% DMSO-treated controls (**A:** A1-A3, 24 h; and **C:** C1-C3, 48 h, show several food vacuoles (V), mitochondria (M), nucleus (N) and lipid droplets (L). Exposure to 0.2 μM posaconazole (**B:** B1-B3, 24 h; **D:** D1-D3, 48 h) led to mitochondrial swelling, accumulation of atypical lipid droplets, alteration of nuclear membrane and appearance of autophagic vacuoles (A) engulfing organelle debris and myelin figures (MF). Bar = 500 nm.

DMSO-treated *N*. *fowleri* trophozoites displayed normal morphology with several food vacuoles, mitochondria, lipid droplets and a nucleus containing one large nucleolus (**Fig 5A**). Lipid droplets serve as the energy and carbon reservoirs in all domains of life. From analysis of another free-living amoeba, *Dictyostelium discoideum*, reported elsewhere,[32] we know that amoeba lipid droplets consist of a hydrophobic core of triglycerides, steryl esters and free sterols surrounded by one leaflet derived from the endoplasmic reticulum membrane to which a specific set of proteins is bound. Between 24 and 48 hours of undisrupted growth, *N*. *fowleri* lipid droplets increased in number and density (**Fig 5C**).

Treatment with posaconazole led to disorganization *of N*. *fowleri* membranes, swelling of mitochondria and appearance of multiple autophagic vacuoles engulfing organelle debris and myelin figures (loops of membranes), indicative of disruption of lipid metabolism. (**Fig 5B and 5D**). Similar ultrastructural alterations were previously reported in *N*. *fowleri* treated with AmpB, including mitochondrial abnormalities and an increase in autophagic vacuoles with myelin-like membranous whorls.[33] AmpB-treated amoebae also had abnormally shaped nucleus and an increase in rough and smooth endoplasmic reticulum membranes,[33] alterations not observed with posaconazole. Finally, lipid droplets of the AmpB-treated amoebae were clustered and enclosed in membranous sheet, whereas treatment with posaconazole in these studies resulted in dispersion of lipid droplets throughout the cell with decrease in size and density.

Consistent with the rate of growth inhibition experiment (**Fig 2**), significant differences in cell ultrastructure are observed after 24 h of posaconazole exposure and no further differences were recorded between 24 h and 48 h of treatment (**Fig 5B vs. Fig 5D**). This observation distinguishes *N*. *fowleri* from kinetoplastid parasites where CYP51 inhibitors are notoriously slow-acting.[34] In *T*. *cruzi*, ultrastructural alterations and increase in autophagic vacuoles are first observed after 72–96 h drug exposure when lipid droplets are largely exhausted.[35, 36] In *N*. *fowleri*, dispersed lipid droplets albeit of reduced density are present after 48 h in significantly damaged *Naegleria* cells. Similar lipid accumulation after posaconazole exposure is observed in *Leishmania amazonensis*, both by TEM and fluorescent staining by Nile Red.[37]

### Interaction of conazoles with the recombinant NfCYP51 target by UV-vis spectroscopy

**Fig 6:**
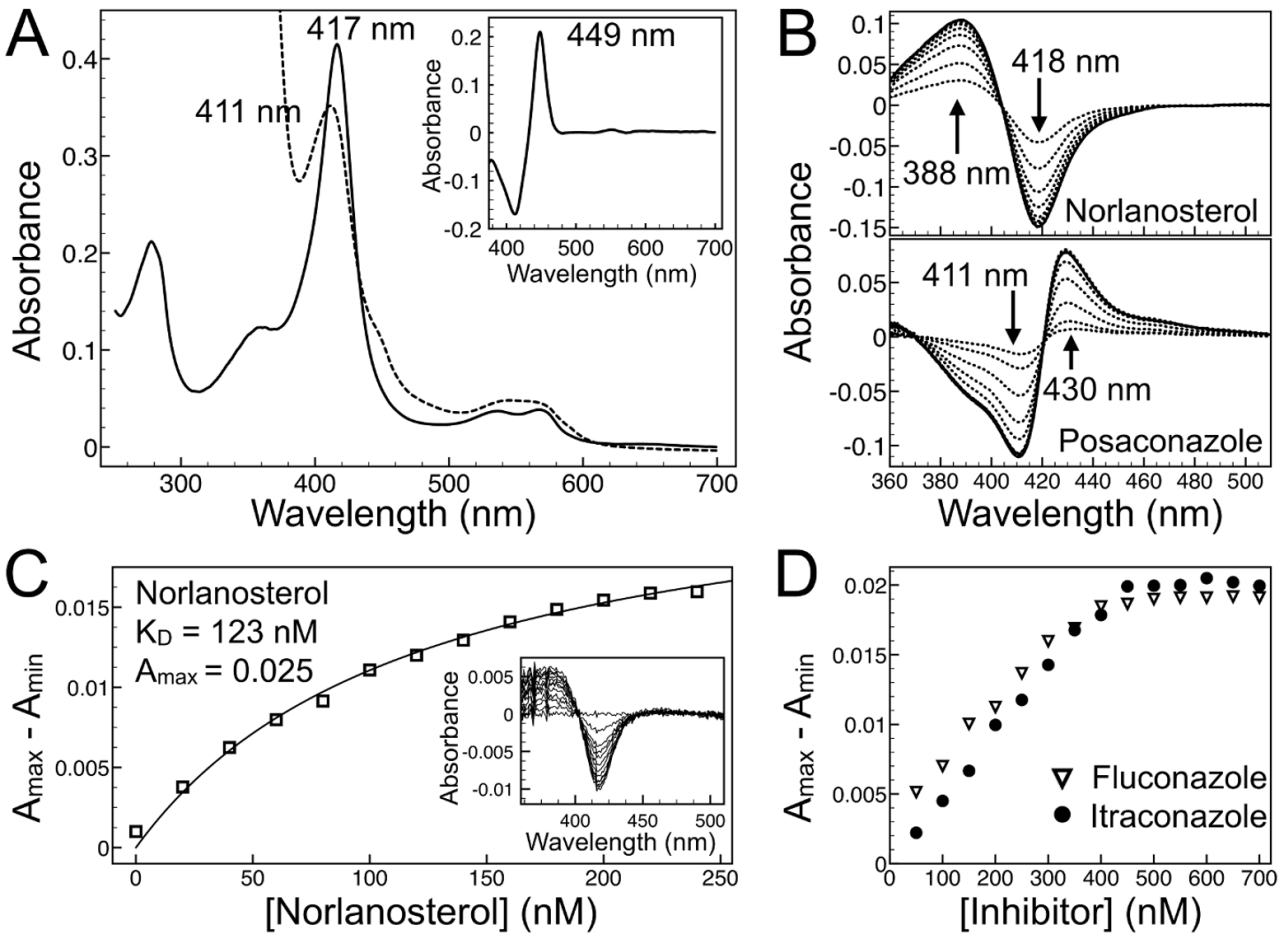
Spectroscopic analysis of recombinant NfCYP51. (**A**) Absolute UV-visible spectra of 3.5 μM purified recombinant NfCYP51: ferric, Fe^3+^,—solid line, ferrous, Fe^2+^,—dashed line. Inset: Fe^2+^- Fe^2+^CO difference spectra. (**B)** Type I (31-norlanosterol) and type II (posaconazole) difference spectra both added in 500 nM increments to 3.5 μM NfCYP51. (**C**) Binding isotherm of 31-norlanosterol added in 25 nM increments to 0.2 μM NfCYP51 and absorbance difference spectra (Inset). (**D**) Binding isotherms of posaconazole and fluconazole, both added in 50 nM increments to 0.5 μM NfCYP51, show a linear increase in signal up until the equivalence point, after which no further increase in signal was detected.

**Fig 7:**
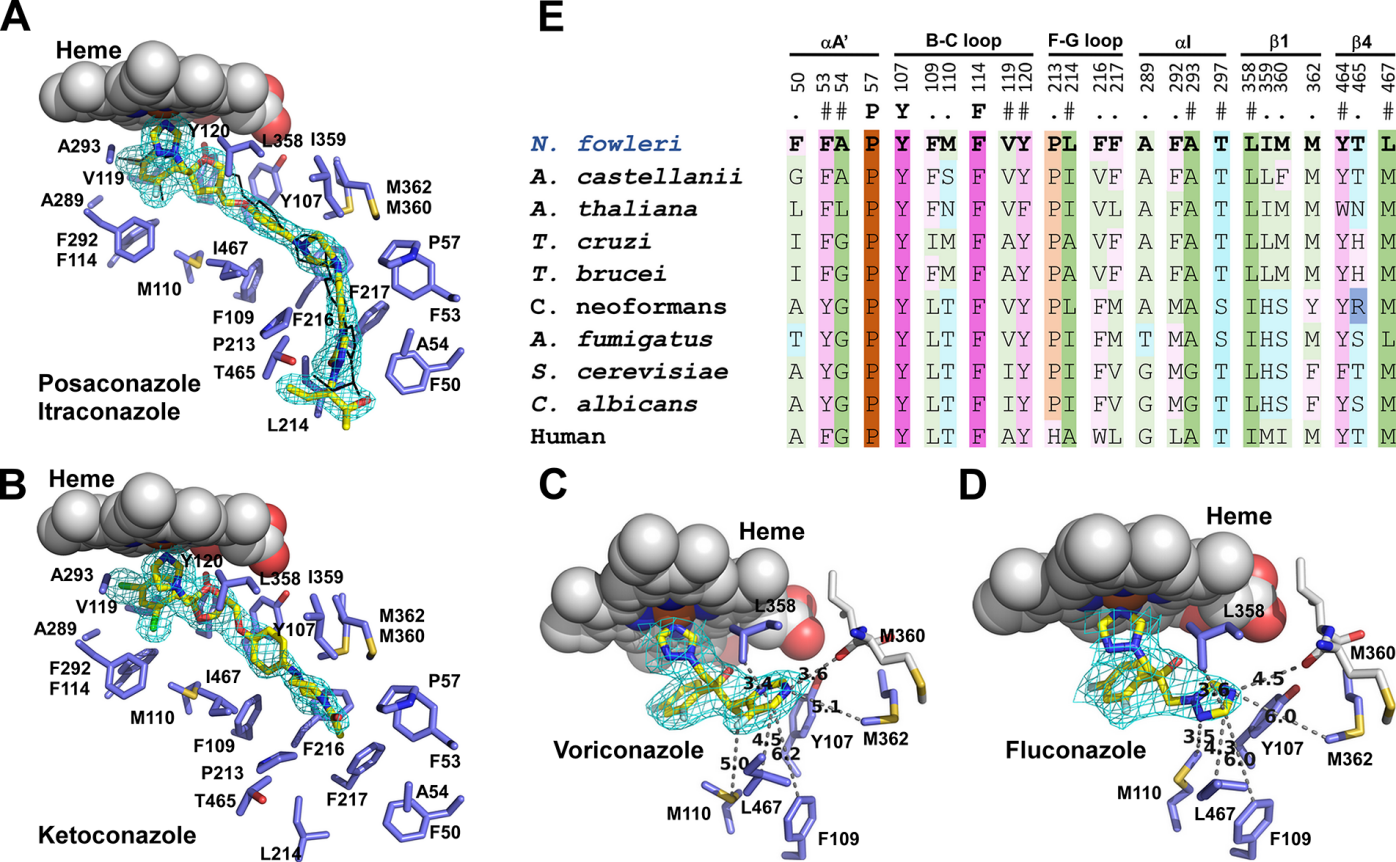
NfCYP51-inhibitor binding. (**A**) Posaconazole (*yellow sticks*) and itraconazole (*black lines*) are shown overlapped. Fragment of the 1.7 Å 2F_o_-F_c_ electron density map countered at 1.σ is shown for posaconazole (*cyan mesh*). Amino acid residues within 5 Å are in *blue*, heme is in *spheres*. Ketoconazole (**B**), voriconazole (**C**) and fluconazole (**D**) are shown in *yellow sticks* with the corresponding map fragments in *cyan mesh*. In **C** and **D**, amino acid contacts only for the inhibitor moiety distant from the heme macrocycle are shown. Distances are in Angstroms. (**E**) Amino acid residues within 5 Å of bound posaconazole in NfCYP51 (highlighted in bold) are propagated to the CYP51 sequences from the indicated species. Residue labeling is according to NfCYP51. Secondary structure elements on the top are labeled according to the nomenclature introduced elsewhere.[47] The color scheme is according to the side chains: hydrophilic neutral (*cyan*), aromatic (*purple*), hydrophobic (*green*) and proline (*ochre*). Residue conservation is indicated by the color shades, with pale → bright gradient corresponds to conservation levels. Invariant positions are in brightest shades.

The recombinant NfCYP51 was authenticated and the drug-target interactions were characterized by UV-vis spectroscopy. The ferric, Fe^3+^, spectrum of NfCYP51 is typical of that of a low-spin P450 (CYP) with a Soret band at 417 nm, while that of the dithionite reduced ferrous, Fe^2+^, species has a Soret band of 411 nm (**Fig 6A**). The 449 nm Soret band of the dithionite reduced and CO bound NfCYP51 is consistent with that of functional CYP enzymes (**Fig 6A**, inset). Upon binding to P450, a heterocyclic drug replaces the heme axial water ligand resulting in a red-shift of the iron Soret band, known as type II.[38] Conazoles bound to NfCYP51 produced type II low-spin difference spectra with a trough at 411 nm and a peak at 430 nm, indicative of azole coordination to the heme iron (**Fig 6B**). 31-Norlanosterol binding produced a type I high-spin difference spectrum with a peak at 388 nm and a trough at 418 nm (**Fig 6B**), resulting from the expulsion of water molecule ligand from the iron coordination sphere by the incoming substrate.[38] The dissociation constant, K_*D*_, of 124±25 nM, was calculated for 31-norlanosterol by fitting plotted spectroscopic data to the standard Michaelis-Menten or Morrison binding equations (**Fig 6C**), both yielding the same K_*D*_ value. The dissociation constants for conazoles could not be calculated by this method due to enzyme saturation reached after the addition of one molar equivalent of a conazole drug to 0.5 μM NfCYP51, as illustrated for fluconazole and posaconazole (**Fig 6D**). When sub-stoichiometric concentrations of drugs were titrated into the enzyme solution, a linear increase in signal was observed for all conazoles up until the equivalence point, after which no further increase in signal was detected. Dilution of NfCYP51 below 0.5 μM resulted in a substantial drop in signal-to-noise ratio in the recorded spectra. From the UV-vis data, we conclude that even the smallest azole drug, fluconazole, binds NfCYP51 with affinity roughly an order of magnitude exceeding that of natural substrate, 31-norlanosterol. The binding superiority of the azole drugs over natural substrate is achieved due to the formation of the coordination bond between an aromatic nitrogen of the azole heterocycle and the heme iron (**Fig 7A–7D**).

### Drug-target interactions by x-ray crystallography

#### Overall structure

To further characterize drug-target interactions, we have screened conazoles for co-crystallization propensity with the NfCYP51 target and determined the co-crystal structures of NfCYP51 bound to five conazole drugs: posaconazole (1.71 Å), ketoconazole (1.87 Å), itraconazole (2.6 Å), voriconazole (2.4 Å) and fluconazole (2.7 Å) (**Table 4**). All conazoles form a coordination bond to the heme iron via an aromatic nitrogen of the heterocycle moiety (**Fig 7A–7D**). The overall protein scaffold of NfCYP51 is similar to that of previously characterized CYP51 from other eukaryotes with the qualification that certain secondary structure elements sample multiple conformations spontaneously or in response to the incoming ligand.[39–41] A number of the co-crystal structures for human,[42] yeast,[43, 44] fungi[45, 46] and kinetoplastid[21] orthologues reported to date point at the BC- and FG-loops and the F- and G-helices (nomenclature introduced by Poulos et al.[47]), as the most conformationally variable structural elements whose concerted motion modulates the size and topology of the CYP51 binding site.

**Table 4:** Crystallographic data collection and refinement statistics.

Protein	NfCYP51	NfCYP51	NfCYP51	NfCYP51	NfCYP51
Ligand Ligand ID	Posaconazole X2N	Itraconazole 1YN	Ketoconazole KKK	Voriconazole VOR	Fluconazole TPF
PDB ID	5TL8	6AYC	6AYB	6AY6	6AY4
**Data collection**					
Space group	C2	C2	C2	C2	C2
Cell dimensions					
*a*, *b*, *c* (Å)	120.0, 55.0, 71.6	120.3, 55.2, 72.4	119.2, 55.3, 71.6	120.2, 55.3, 73.2	122.1, 55.3, 73.5
*α*, *β*, *γ* (°)	90.0, 100.1, 90.0	90.0, 100.6, 90.0	90.0, 100.1, 90.0	90.0, 100.8, 90.0	90.0, 101.5, 90.0
Molecules in AU	1	1	1	1	1
Wavelength	1.11587	1.11587	1.11587	1.11587	1.11587
Resolution (Å)	1.71	2.60	1.87	2.40	2.70
*R*_*f*_ (%)	4.1 (144.8)^a^	13.3 (286.5)	20.3 (188.7)	10.9 (136.0)	7.0 (160.4)
*I*/σ*I*	14.4 (0.62)	7.1 (0.59)	3.79 (0.53)	7.0 (0.47)	7.94 (0.57)
Completeness (%)	98.3 (85.6)	99.7 (99.8)	95.1 (68.4)	97.3 (66.5)	97.7 (99.8)
Redundancy	3.6 (2.4)	6.5 (6.9)	6.0 (4.7)	5.5 (2.2)	2.6 (2.6)
**Refinement**					
No. reflections	47085	13616	34450	17255	12443
*R*_work_ / *R*_free_ (%)	19.5/24.9	21.8/29.0	24.3/28.4	21.9/29.0	25.7/31.3
No. atoms	3799	3731	3712	3660	3670
Wilson	36.1	86.0	42.9	82.1	85.6
Mean B value	39.9	95.1	46.0	85.3	97.8
R.m.s deviations					
Bond lengths (Å)	0.018	0.011	0.019	0.009	0.008
Bond angles (°)	2.002	1.938	1.260	1.005	1.506

^a^Values for highest-resolution shells are in parentheses

#### Drug-target interactions

Both posaconazole and itraconazole (MW ~700 g/mol) occupy the whole length of the CYP51 hydrophobic tunnel spanning from the heme macrocycle to the protein surface (**Fig 7A**). At Pro213, the tunnel turns forcing the inhibitor’s long moiety to bend at piperazine ring. The bent conformation of posaconazole has been previously reported in *T*. *brucei* CYP51.[48] As evidenced by the F_o_-F_c_ electron density map (**S2 Fig**), the 2,4-dichlorophenyl moiety of itraconazole may flip in the vicinity of heme adopting two alternative conformations. Flipping of the 2,4-difluorophenyl moiety was previously observed in fluconazole.[48] The smaller ketoconazole (MW 531 g/mol) also extends into the hydrophobic tunnel but only to reach Pro213 (**Fig 7B**). Finally, voriconazole and fluconazole (MW 300–350 g/mol), bind in vicinity of heme macrocycle and benefit very little from the tunnel space (**Fig 7C and 7D**). Well-defined by electron density, the 5-fluoropyrimidinyl moiety of voriconazole makes a series of contacts within 6 Å of Tyr107, Phe109, Met110, Leu358, Met362 and Leu467, plus a H-bond to carbonyl oxygen of M360 (3.6 Å) (**Fig 7C**). The smaller and more hydrophilic 1,2,4-triazolyl group of fluconazole is less favored by this environment, as evidenced by the longer distances and the electron density map progressively less defined for the triazole moiety not involved in the coordination of heme iron (**Fig 7D**).

#### Variability of the CYP51 binding site

The first-tier contacts within 5 Å of inhibitors deduced from the NfCYP51 structure have been projected to the CYP51 sequences of other human pathogens (**Fig 7E)**. Predominantly aliphatic and aromatic amino acid residues delineate the binding tunnel in CYP51, with only three of them being invariant across the species. Variable positions with different degree of conservation confer substrate- and inhibitor-binding specificity to the CYP51 orthologues. Consistent with the biochemical data, phylogenetic analysis points at ‘plant-like’ substrate specificity of NfCYP51 defined by Phe109 (numbering is according to NfCYP51) that is only present in the orthologues converting the 4α-monomethylsterol substrates, obtusifoliol or 31-norlanosterol. In this regard, *Naegleria*, *Acanthamoeba* and *T*. *brucei* group together with plant CYP51 represented in **Fig 7E** by *Arabidopsis thaliana*. The *T*. *cruzi*, fungal and mammalian CYP51 having leucine/isoleucine at 109 metabolize 4α, β-dimethylated sterol substrates, lanosterol, eburicol and 24,25-dihydrolanosterol.

Another critical sequence variability is mapped to proline 213. A non-conserved proline-to-histidine substitution in human CYP51 accounts for the host-pathogen selectivity of conazoles. The histidine moiety protruding into the hydrophobic tunnel of human CYP51 interferes with a long substituent of the high MW conazoles. The substantial genetic divergence between the etiological agents of human diseases require tailored implementation of pathogen-specific drug discovery programs utilizing methodologies specific to the targeted pathogens. For *N*. *fowleri*, the crystal structures determined in this work may be indispensable for designing molecules combining potency against NfCYP51 with brain permeability.

While UV-vis spectroscopy could not prioritize conazoles by binding affinity due to insufficient sensitivity of the experimental setup, the co-crystal structures unambiguously link the decline in conazole’s potency to the decrease in a number of drug-target interactions as a function of MW. The highest MW conazoles, posaconazole and itraconazole, making a large number of interactions in the CYP51 hydrophobic tunnel, are broad spectrum. Fluconazole, with the least number of drug-target contacts, is a narrow spectrum antifungal agent with the activity limited to *Candida albicans* (but not *C*. *krusei or C*. *glabrata*) and *Cryptococcus neoformans*. Despite of being part of the drug formulation used for the treatment of PAM patients, fluconazole has the lowest activity against *N*. *fowleri* (EC_50_ of 13.9 μM) among azole drugs. Differences in the first-tier residues directly contacting fluconazole in *N*. *fowleri* and *C*. *albicans* (**Fig 7E**) may account for the differential activity. Also, high glycine content (positions equivalent to Ala54, Ala289 and Ala293 in *N*. *fowleri* are taken by glycine in *C*. *albicans*) may render *C*. *albicans* CYP51 more adaptive to fluconazole.

### Perspectives of conazoles in the treatment of PAM

Although the potency of some conazoles against cultured *N*. *fowleri* was equal or exceeded that of AmpB (**Table 1**), to attain parasitological cure in PAM patients, a drug must cross the blood-brain barrier (BBB). Conazoles exhibit variable physicochemical characteristics and differ with regard to cerebrospinal fluid (CSF) and brain parenchymal penetration.[30, 49] Across the board, brain permeability of conazoles is inversely related to the MW and to their *in vitro* anti-*Naegleria* potency. Thus, fluconazole, having the lowest activity against *N*. *fowleri*, is known to rapidly distribute through body tissues, including different CNS compartments, where it achieves concentrations greater than MIC_90_ of common fungal pathogens.[49, 50] The intermediate MW conazoles penetrate CSF and brain tissue to a different extent. Voriconazole brain permeability has been reported in human studies of meningitis patients.[51–53] Miconazole is effective in treatment of human fungal meningitis not susceptible to AmpB; high brain concentrations of miconazole are achieved by intrathecal (IT) administration.[54, 55] Rabbit studies have shown that ravuconazole[56] and ketoconazole[57] penetrate brain tissue; intermediate concentrations of ketoconazole were found in CSF and are modestly increased in the presence of meningeal inflammation.[57]

Finally, the highest MW conazoles—posaconazole and itraconazole–have CNS pharmacokinetics similar to that of a cornerstone of PAM therapy, AmpB. In rabbit studies, none of the AmpB formulations produced measurable concentrations in the CSF regardless of CNS inflammation.[58, 59] At the same time, detectable brain parenchymal AmpB concentrations were observed even in the absence of CNS infection (3–27% of serum concentrations) and increased two- to four-fold in presence of infection.[59] Animal model and human studies reported nearly undetectable CSF concentrations of itraconazole,[60–62] while posaconazole showed striking differences in the CSF-to-plasma ratios ranging from the below limit of detection to 2.4.[63] Disturbance of the BBB tight junctions by inflammation may facilitate passage of posaconazole into the CSF.[64] Despite the lack of appreciable CSF concentrations, itraconazole accumulates in brain parenchyma as an active hydroxylated metabolite at concentrations higher than the MIC of the infecting fungi.[65] The ability of itraconazole and posaconazole to accumulate in brain tissue may account for successful use of these drugs for CNS invasive fungal infections in humans.[57, 66–68] If brain parenchymal kinetics are valid predictor of antifungal efficacy in the treatment of CNS mycoses compared to CSF concentrations,[49] the same may be true for anti-*Naegleria* efficacy. Systematic assessment of conazoles in an animal model of PAM would single out the most efficacious drug of this class for the treatment of PAM.

#### Summary

Based on the collective evidence of the target-based and whole-parasite studies, we conclude that amoebicidal effect of conazoles in *N*. *fowleri* is due to depletion of the ergosterol pool concomitant with accumulation in large amounts of sterol intermediates with molecular structure/physicochemical properties incompatible with normal cell physiology. Disruption of CYP51 function induces massive autophagocytosis leading to rapid *N*. *fowleri* cell death. These data validate CYP51 as an essential enzyme and potentially a druggable target in *N*. *fowleri*. However, the amoebicidal activity of conazoles is inversely related to their brain permeability: it increases with the increase of drug molecular weight (MW), reaching low nanomolar potency for posaconazole and itraconazole. The low anti-*N*. *fowleri* activity of the brain-penetrant fluconazole questions its role in a drug combination currently recommended by CDC for the PAM treatment.

## Materials and methods

### Materials

The *Naegleria fowleri* strain KUL originally isolated from human cerebrospinal fluid in Belgium in 1973[69] was obtained from ATCC. KUL is type 3 strain based on the length of the internal transcribed spacers 1 (ITS1), with the T at position 31 in the 5.8S rDNA sequence.[3]

Azole inhibitors were purchased from commercial sources: fluconazole from Cayman Chemical, clotrimazole, miconazole (racemic mix) and voriconazole from Sigma-Aldrich, ketoconazole and itraconazole from Alfa Aesar, voriconazole and isavuconazole from StruChem (China). Posaconazole was purified from a Noxafil (Merck) suspension purchased from a pharmacy, as previously described.[35] Miltefosine and amphotericin B were purchased from Sigma-Aldrich. Sulfobutylether-β-cyclodextrin (SBE-β-CD), also known as Captisol, was from MedChem Express and methyl-β-cyclodextrin (M-β-CD) was from Sigma-Aldrich. 31-Norlanosterol was purified from *Candida albicans* treated with both CYP51 and SMT inhibitors; the structure of the sterol was authenticated by both the GC-MS and NMR methods.

Drug stock solutions were freshly made either in DMSO or 40% SBE-β-CD. The DMSO stock was used for preparation of the electron microscopy and GC-MS samples, while the SBE-β-CD stock was used for serial dilutions in the growth inhibition experiments. The SBE-β-CD stock solutions were prepared based on the compositions for the posaconazole intravenous administration provided in the US Patent 2013/0096053 A1.[31]. Briefly, a 40% solution of SBE-β-CD was prepared by dissolving 4 grams of SBE-β-CD in 10 ml of ddH_2_O. Five μmoles of each drug was added to 1 ml of 40% SBE-β-CD and sonicated until dissolved. In order to solubilize posaconazole and itraconazole, the drug-SBE-β-CD suspension was acidified to pH 2 by the addition of 15% HCl and then sonicated.

### Validation of azole inhibitors for amoebicidal activity

To determine EC_50_ values, conazoles were tested for dose-response against *N*. *fowleri* trophozoites axenically cultured in Nelson’s medium supplemented with 10% fetal bovine serum at 37°C;[70] all the experiments were performed in triplicate using trophozoites harvested during the logarithmic phase of growth.[71] Drug concentration ranges of 0.4–50 μM and 0.008–25 μM in 0.2% SBE-β-CD were generated by transferring 0.5 μl of serially diluted compounds to a corresponding well of the 96-well plate followed by addition of 99.5 μl of *N*. *fowleri* trophozoites (10,000 amoebae). Assay plates were incubated for 48 h and cell viability was determined by the CellTiter-Glo Luminescent Cell Viability Assay.[26, 71] The experiments using trophozoites were conducted in a biosafety cabinet following the BSL2 procedures as specified in the UCSD Biosafety Practices Guidelines.

### Ultrastructural analysis by transmission electron microscopy (TEM)

*N*. *fowleri* trophozoites (2x10^6^) were treated with posaconazole at 0.2 μM, for 24 h and 48 h, washed with PBS and then fixed overnight at 4°C in modified Karnovsky’s fixative (2.5% glutaraldehyde and 2% paraformaldehyde in 0.1 M sodium phosphate, pH 7.2).[72] 0.1% DMSO-treated controls were simultaneously processed. The samples were then post-fixed for 1 hour with 1% osmium tetroxide in 0.15 M sodium cacodylate, pH 7.4, dehydrated with an ascending series of ethyl alcohol and propylene oxide, and finally embedded in an Epon resin (Scipoxy 812, Energy Beam Sciences). Thin sections (50–60 nm) were cut using Leica UCT ultramicrotome, mounted on the Formvar and carbon-coated copper grids, and counterstained for 5 min with 2% uranyl acetate followed by Sato's lead stain for 1 min. *Naegleria* thin sections were examined using a Tecnai G2 Spirit BioTWIN transmission electron microscope (TEM) equipped with an Eagle 4k HS digital camera (FEI, Hilsboro, OR).

### GC-MS analysis of the *Naegleria* sterols

*N*. *fowleri* sterols were analyzed by the use of GC-MS, wherein the lipids extracted from *N*. *fowleri* trophozoites grown in the presence of a vehicle or inhibitor at concentrations that produce ultrastructural changes without destroying a parasite cell, were separated by gas chromatography and subsequently analyzed by electron-ionization mass-spectrometry (EI). 2x10^7^ or 5x10^7^ trophozoites per sample were treated with 0.1% DMSO alone or 0.2 μM posaconazole dissolved in DMSO. AmpB, a drug with a different mechanism of action, was used as a negative control at 0.2 μM. To avoid parasite death, drug exposure was terminated after 24 h when the amoebae were pelleted by centrifugation, washed three times with PBS (3x10 ml) and, finally, 2 ml of chloroform/methanol 2:1 solution was added to the cell pellet. The organic solvents were evaporated under N_2_ flow, and the pellet was incubated for 24 h with 3 ml chloroform. Polar molecules were removed by several extractions with water (3x10 ml). The organic solvent was then subsequently changed to chloroform/methanol 9:1 and then acetonitrile (3 ml each) through evaporation under N_2_ flow; each step followed by triple washes in water as described above.

Extracted sterols were either directly analyzed as free sterols, or first derivitized with TMS group. For free sterol analysis, extracted dry sterols were re-dissolved in chloroform (100 μl) and 2 μl of each sample were injected into the analytical column of the Agilent 6890 gas chromatograph (the inject port temperature was controlled at 250°C), coupled to a 5973 mass selective detector (MSD). The sterols were separated using a ZB5 capillary column (30m X 250um X0.25um) with helium carrier gas flow rate set at 1.2 ml/min and temperature profile beginning at 170°C for 1 min, then increased by 20°C/min to 280°C, and then hold at 280°C for 20 min. The mass spectrometer scanned m/z 50−500 during the course of analysis. The sterols were identified by comparing the GC retention time to that of the internal cholesterol[73] and the fragmentation patterns to that of the authentic standards and the NIST (2008) mass spectral library. A forward and reverse match score of 800 and above was considered a correct match. The sterols were quantified based on the total ion current peak areas of each sterol.

For chemical derivatization, extracted dry sterols were dissolved in 30 μl of hexane and 70 μl of N,N-bis(trimethylsilyl)-2,2,2-trifluoroacetamide (BSTFA), and incubated for 2 h at 37°C. Three microliters of the TMS-derivatized lipid mixture was injected directly into an Agilent 7820A gas chromatography system coupled to a mass selective detector. The inject port temperature was controlled at 250°C, the helium carrier gas flow rate was set at 13 ml/min. The lipids were separated on the analytical column using a temperature profile that begins at 200°C for 3 min, increases by 15°C/min to 270°C, and then holds at 270°C for 30 min, finishing with post run 280°C 4 min. The mass spectrometer scanned m/z 50−750 during the course of analysis.

### NfCYP51 expression and purification

NfCYP51, codon-optimized for bacterial expression, had a coding sequence with 34 N-terminal membrane anchoring residues replaced with the MAKKTSSKGKL to increase recombinant protein recovery during purification. (**S1 Data**). This construct was generated synthetically (GenScript) and cloned into the pCW-LIC expression vector obtained from the non-profit plasmid repository (Addgene, Cambridge, MA). NfCYP51 was expressed in DH5α *E*. *coli* strain co-transformed with the pGro7 plasmid (Takara) carrying GroEL/ES chaperones with induction by 0.5 mM isopropyl-β-D-thiogalactopyranoside (IPTG) for 40–48 hours at 25°C. All purification steps were carried out at 4°C. Cells were pelleted, re-suspended in the lysis buffer (50 mM K-PO4, pH 8.0; 100 mM NaCl, 10% glycerol, 1 mM EDTA, 1 mM DTT and 0.5 mM PMSF) and then disrupted using the fluid processor Microfluidics M-110P (Microfluidics Inc.). Non-ionic detergent CHAPS was added to 0.5% and cell lysate was incubated for 30 min prior to centrifugation. The crude extract was separated from cell debris. Cleared lysate was loaded to a Ni-NTA column and after a series of washes NfCYP51 was eluted by increasing the imidazole concentration from 0 to 500 mM. Fractions containing NfCYP51 were pooled and passed through Q-Sepharose and then S-Sepharose (GE Healthcare Life Sciences). NfCYP51 flowed-through both ion exchange columns was then bound to hydroxyapatite (HAP) column (BioRad) and eluted from it in the gradient of K-PO_4_ concentrations from 0.02 M to 0.8 M supplemented with 10% glycerol, 0.5 mM EDTA and 1 mM DTT. Fractions containing pure NfCYP51 were pooled, concentrated, aliquoted and frozen at -80°C.

### UV-vis spectroscopy of NfCYP51

Recombinant NfCYP51 was characterized spectrally for integrity of the heme prosthetic group and for substrate and inhibitor binding. All spectra were recorded using a Cary 1E (Varian) dual beam UV-visible spectrophotometer. Purified NfCYP51 was diluted to 3.5 μM in 50 mM K-PO_4_ (pH 7.4) and 10% glycerol buffer and allowed to equilibrate to room temperature for 10 min prior to readings. Spectra were recorded from 250–700 nm for the ferric and dithionite reduced ferrous NfCYP51 with buffer in the reference cuvette. The CO difference spectrum was recorded by splitting dithionite reduced ferrous NfCYP51 into the sample and reference cuvettes. A baseline was recorded and CO was bubbled into the sample cuvette, after which the difference spectrum was recorded. The concentration of NfCYP51 was calculated using the extinction coefficient ε_450_ = 91 mM^-1^cm^-1^.

Spectral binding titrations were performed at 25°C using 0.5 μM NfCYP51 for conazole and 0.2 μM for 31-norlanosterol. All conazoles with the exception of itraconazole were dissolved in 40% SBE-β-CD and then diluted to 100 μM stocks in 0.8% SBE-β-CD. Itraconazole was dissolved in DMSO. 31-Norlanosterol was dissolved in 20% M-β-CD and diluted to 100 μM. For each titration 2 ml of NfCYP51 was split equally for the reference and sample cuvettes with ligand or inhibitor being added to the sample cuvette while vehicle alone was added to the reference cuvette, with the total added volume being less than 1% of the total volume. Spectra were recorded from 350 to 500 nm. A binding isotherm for 31-norlanosterol was generated by plotting the absorbance minimum subtracted from the absorbance maximum as function of drug concentration. The spectral dissociation constant K_D_ was estimated using the Curve Fitting Tool in MATLAB (MathWorks, Natick, MA) by fitting the binding isotherm using the hyperbolic Michaelis-Menten equation ΔA = ΔA_max_[L]/(K_D_+[L]) or the quadratic Morrison equation ΔA = (ΔA_max_ / 2[E])((K_D_ + [L] + [E])—((K_D_ + [E] + [L])^2^–4[E][L])^0.5^) where ΔA is the difference between absorbance maximum and minimum, ΔA_max_ is the extrapolated maximum absorbtion difference, [L] is the ligand concentration and [E] is the enzyme concentration.

### Crystallization and x-ray structure determination

Prior to crystallization, NfCYP51 was diluted to 0.5 mM by mixing with 0.6 mM inhibitor added to the desired volume of water from the 10 mM or 100 mM stock solutions in DMSO, depending on compound solubility. Screening of crystallization conditions for each inhibitor complex was performed using commercial high-throughput screening kits available in deep-well format (Hampton Research or Qiagen), a nanoliter drop-setting Mosquito robot (TTP LabTech) operating with 96-well plates, and a hanging drop crystallization protocol. For diffraction quality, crystals were further optimized in 96-well plates configured using the Dragonfly robot (TTP LabTech) and the Designer software (TTP LabTech). All crystals were harvested from the narrow grid of crystallization conditions: 30–33% PEG MME 550, 30 mM CaCl_2_, 0–4% Jeffamine M-600, 0.1 M bis-Tris propane, pH 7.1–7.5.

Diffraction data were collected at 100–110 K at beamline 8.3.1, Advanced Light Source, Lawrence Berkeley National Laboratory, USA. Data indexing, integration, and scaling were conducted using XDS.[74] The high-resolution crystal structure of the NfCYP51-posaconazole complex was determined by molecular replacement using as a search model *T*. *cruzi* CYP51, PDB ID 2X2N. The initial model was built using the BUCCANEER[75, 76] and COOT[77] programs. Refinement was performed by using REFMAC5 software.[76, 78] The newly determined crystal structure (PDB ID 5TL8) was subsequently used for other NfCYP51-conazole complexes reported in this work. Data collection and refinement statistics are shown in **Table 4**.

#### Accession codes

The atomic coordinates and structure factors (5TL8, 6AY4, 6AY6, 6AYB and 6AYC) have been deposited in the Protein Data Bank, Research Collaboratory for Structural Bioinformatics, Rutgers University, New Brunswick, NJ (http://www.rcsb.org/)

## Supporting information

S1 Data Recombinant NfCYP51. NfCYP51 codon-optimized DNA sequence synthetically generated (GenScript, Piscataway, NJ) for bacterial expression—with 34 N-terminal residues replaced with the MAKKTSSKGKL leading sequence (to inprove protein expression and purification)—and cloned into the pCW-LIC expression vector obtained from the non-profit plasmid repository (Addgene, Cambridge, MA).
(DOCX)

S1 Fig Gas chromatography separation of the total sterol fractions extracted from *N*. *fowleri* trophozoites. Chromatogram fragments from the TMS-derivatized DMSO-, posaconazole- and Amphotericin B-treated *N*. *fowleri* lipid extracts are shown. Peaks are labeled according to **Table 2**. The sterol identities were assigned based on relative chromatographic behavior, the characteristic molecular masses and electron ionization (EI) fragmentation patterns by comparing them to the authentic standards and the NIST (2008) mass spectral library. In contrast to posaconazole, Amphotericin B, a drug with a different mechanism of action used as a negative control, did not perturb the native sterol pattern.
(TIF)

S2 Fig Flipping of the 2,4-dichlorophenyl moiety of itraconazole evidenced by electron density. Itraconazole in a single confirmation (*yellow sticks*) is shown in the fragment of the 2.6 Å 2F_o_-F_c_ electron density map countered at 1.0 σ (*cyan mesh*) overlapped with a fragment of the F_o_-F_c_ electron density countered at -3.0 σ (*red mesh*). “Negative” peak at 2-chloro-substituent suggests a possibility of partial occupancy of this site due to flipping of the 2, 4-dichlorophenyl moiety of itraconazole. Heme is shown in van der Waals spheres. Heteroatoms are colored according chemical elements: oxygen–red, nitrogen–blue, chlorine–green, iron–ochre.
(PNG)
