# Supplementary material for: CYP51 is an essential drug target for the treatment of primary amoebic meningoencephalitis (PAM)
Source: PLoS Negl Trop Dis. 2017 Dec 28;11(12):e0006104. doi: 10.1371/journal.pntd.0006104 (PMC5746216; doi:10.1371/journal.pntd.0006104)
Supplement: S1 Data — NfCYP51 codon-optimized DNA sequence synthetically generated (GenScript, Piscataway, NJ) for bacterial expression—with 34 N-terminal residues replaced with the MAKKTSSKGKL leading sequence (to inprove protein expression and purification)—and cloned into the pCW-LIC expression vector obtained from the non-profit plasmid repository (Addgene, Cambridge, MA). (DOCX) [file pntd.0006104.s001.docx]

**NFCYP51 amino acid sequence:**

MAKKTSSKGKLPPRVPNLIPYVGSFVSFAKNPVQFIIDNSKKYGDVFTATILGKEMTFLNHPKILDTFFKATDNELSLRDVYRFMRPVFGTGVVYDADSTERMMEQVKFVSSGLTTARFRVFVDIFEDEIAHKVKELGPEGTVDVAELMADLIIFTASRCLLGDEVRQYLSEKNLGKLYHDIDDGISPLSFFYPSLPAPKRDKARKAVGEIFQELLDKRREEHKKHPERLLDESKMDVVDHLLTQKYKDGQELTDVHRIGILIAGLFAGQHTSSITSSWTLMNVISNKKVLEKVRKEQEEIMGSDKVLDYDKVMKMDYLEACMKEALRMYPPLIMIMRMARKPRECEQYIIPKGNILVVSPSVAGRCTDTYTNPDVFDPERLTERKEHEKFKYGAVPFGAGRHKCIGENFALLQVKSIISILLRYFDMEYIGKIPDPSYTSLVVGPSPPTRMRYKLRKQQHHHHHH

**Codon-optimized DNA sequence:**

ATGGCTAAAAAGACATCTTCAAAAGGTAAACTGCCACCACGTGTTCCAAATCTGATTCCTTATGTCGGCTCCTTTGTATCGTTCGCTAAAAATCCGGTCCAGTTTATTATCGATAACAGCAAAAAGTACGGTGACGTTTTTACCGCAACGATCCTGGGCAAAGAAATGACTTTCTTGAATCATCCGAAGATTCTGGATACATTTTTCAAAGCTACGGACAACGAACTGTCCTTAAGAGATGTTTATCGTTTTATGAGACCTGTGTTCGGTACTGGTGTTGTGTACGATGCAGACTCGACTGAACGCATGATGGAACAGGTTAAATTTGTGAGCAGTGGTTTAACCACTGCCCGCTTTCGTGTTTTCGTTGATATTTTCGAAGACGAAATTGCGCATAAAGTTAAGGAACTGGGTCCTGAAGGCACAGTTGATGTAGCCGAATTAATGGCGGATTTGATTATCTTTACGGCCTCTCGTTGTCTGTTAGGCGATGAAGTGAGACAATATCTGTCAGAAAAGAACTTGGGTAAACTGTACCACGATATTGATGACGGCATTTCTCCTTTGTCATTTTTCTATCCATCCCTGCCAGCGCCGAAACGCGATAAGGCTCGTAAAGCAGTTGGCGAAATTTTTCAGGAATTGCTGGATAAACGTAGAGAAGAACATAAAAAGCACCCAGAACGTTTATTGGATGAATCAAAAATGGATGTCGTAGACCATCTGCTGACACAAAAATATAAGGATGGTCAGGAACTGACGGACGTGCATAGAATTGGCATCCTGATTGCTGGTTTATTCGCAGGCCAACACACGTCCTCGATCACCTCTTCATGGACTTTGATGAACGTTATTAGCAACAAAAAGGTTTTGGAAAAGGTGCGCAAAGAACAGGAAGAAATTATGGGTAGTGACAAAGTCCTGGATTACGACAAGGTAATGAAGATGGATTACTTAGAAGCCTGTATGAAAGAAGCGTTACGTATGTATCCTCCATTGATCATGATTATGCGCATGGCCCGTAAGCCGAGAGAATGTGAACAATACATCATCCCTAAGGGTAACATTCTGGTTGTGAGCCCAAGTGTTGCGGGCAGATGCACCGATACTTATACAAACCCAGATGTGTTTGACCCGGAAAGACTGACCGAACGCAAAGAACATGAAAAATTCAAATACGGTGCTGTCCCATTCGGTGCAGGCCGCCACAAGTGCATCGGCGAAAATTTTGCTTTGCTGCAGGTTAAGAGCATCATCAGTATCTTGTTGCGTTACTTCGACATGGAATACATCGGTAAAATTCCTGATCCATCCTATACCTCGTTGGTCGTAGGCCCGTCTCCGCCTACTAGAATGCGCTACAAGCTGCGCAAACAACAGCATCACCATCACCATCACTAA
