# Supplementary figures and images for: CYP51 is an essential drug target for the treatment of primary amoebic meningoencephalitis (PAM)
Source: PLoS Negl Trop Dis. 2017 Dec 28;11(12):e0006104. doi: 10.1371/journal.pntd.0006104 (PMC5746216; doi:10.1371/journal.pntd.0006104)

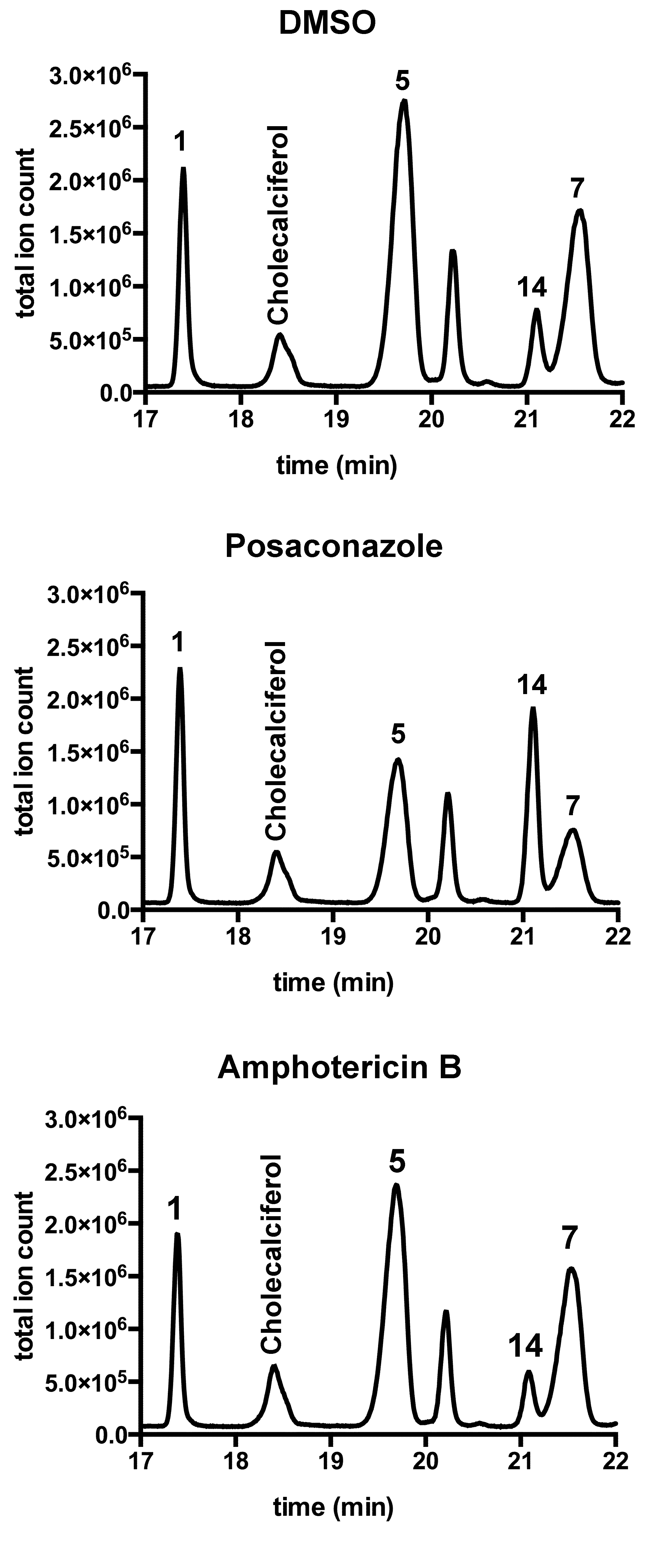

Supplement: S1 Fig — Chromatogram fragments from the TMS-derivatized DMSO-, posaconazole- and Amphotericin B-treated N. fowleri lipid extracts are shown. Peaks are labeled according to Table 2. The sterol identities were assigned based on relative chromatographic behavior, the characteristic molecular masses and electron ionization (EI) fragmentation patterns by comparing them to the authentic standards and the NIST (2008) mass spectral library. In contrast to posaconazole, Amphotericin B, a drug with a different mechanism of action used as a negative control, did not perturb the native sterol pattern. (TIF) [file pntd.0006104.s002.tif]

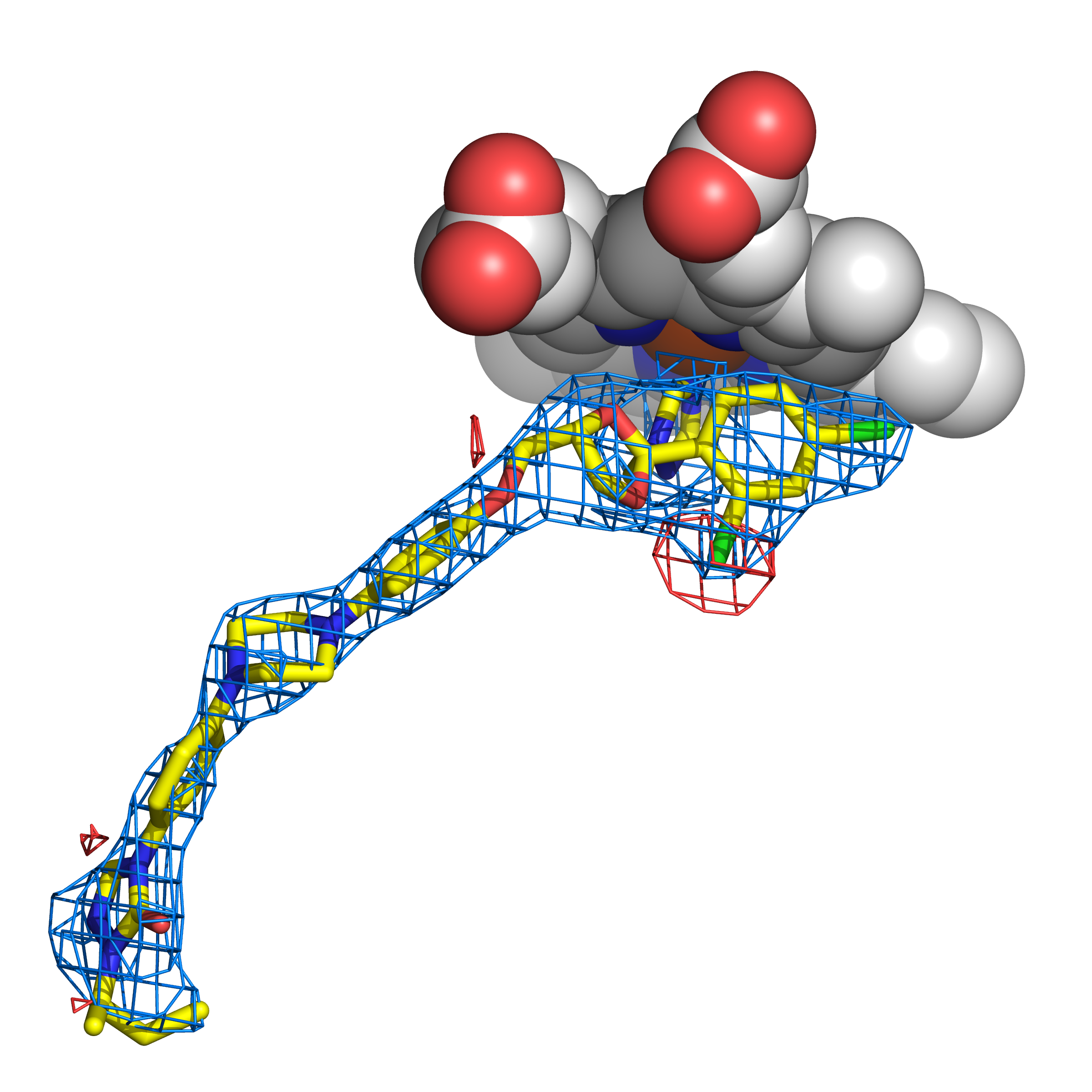

Supplement: S2 Fig — Itraconazole in a single confirmation (yellow sticks) is shown in the fragment of the 2.6 Å 2Fo-Fc electron density map countered at 1.0 σ (cyan mesh) overlapped with a fragment of the Fo-Fc electron density countered at -3.0 σ (red mesh). “Negative” peak at 2-chloro-substituent suggests a possibility of partial occupancy of this site due to flipping of the 2, 4-dichlorophenyl moiety of itraconazole. Heme is shown in van der Waals spheres. Heteroatoms are colored according chemical elements: oxygen–red, nitrogen–blue, chlorine–green, iron–ochre. (PNG) [file pntd.0006104.s003.png]
